# The Utility of a Mental Health App in Apprentice Workers: A Pilot Study

**DOI:** 10.3389/fpubh.2020.00389

**Published:** 2020-09-04

**Authors:** Mark Deady, Nicholas Glozier, Daniel Collins, Rochelle Einboden, Isobel Lavender, Alexis Wray, Aimee Gayed, Rafael A. Calvo, Helen Christensen, Samuel B. Harvey, Isabella Choi

**Affiliations:** ^1^Faculty of Medicine, Black Dog Institute, UNSW, Sydney, NSW, Australia; ^2^Faculty of Medicine and Health, Brain and Mind Centre, Central Clinical School, The University of Sydney, Sydney, NSW, Australia; ^3^Faculty of Medicine and Health, Susan Wakil School of Nursing and Midwifery, The University of Sydney, Sydney, NSW, Australia; ^4^SafeWork NSW, Sydney, NSW, Australia; ^5^WorkSafe ACT, Canberra, ACT, Australia; ^6^Dyson School of Design Engineering, Imperial College London, London, United Kingdom

**Keywords:** mental health, apprentice, workplace, depression, eHealth, smartphone, feasibility, pilot

## Abstract

**Background:** Young people are at heightened risk for mental health problems. Apprenticeships are common pathways into the workforce at a critical developmental period. However, in some cases the working conditions for apprentices present significant challenges to mental wellness. As apprentices are unlikely to utilize traditional services, eHealth and mHealth interventions are a useful means of delivering interventions to this group. The aim of the current paper is to: (1) qualitatively explore the utility of an existing mental health app within an apprentice population; and (2) evaluate the usability, acceptability, feasibility and preliminary efficacy of a modified version of the app (*HeadGear Apprentice*), designed to reduce depressive symptoms in an apprentice working population.

**Methods:** Study One: Twenty-six apprentices (aged 18–30) took part in one of eight (90-min) focus groups. Participants explored the *HeadGear* app, took part in group discussions, and completed uMARS questionnaires. Modifications were made to the app based on feedback. Study Two: In the follow-up pilot testing, 47 apprentices downloaded and used the modified app over 30 days. Assessment occurred online at baseline, 5-weeks, and 3-months post-baseline. Feasibility was evaluated using consent rates, adherence and attrition. Acceptability was assessed using questionnaires and a post-study interview. Depression, anxiety, well-being, and work performance scores were used to assess preliminary efficacy.

**Results:** Overall, the app was well-received in both studies, with high self-reported scores for acceptability and utility. However, engagement—both in terms of self-report and adherence—was an issue in both studies. In Study Two, users completed approximately one-third of the app's therapeutic content, with increased usage associated with improved outcomes. This had implications for the preliminary effectiveness of the app [depression as measured by the PHQ-9 *Cohen's d* = 0.27 (95%CI:-0.16–0.69)]. At follow-up users reported improvements in all outcomes, but overall only change in well-being reached statistical significance [*Cohen's d* = −0.29 (95%CI:-0.72–0.14)].

**Conclusion:** Overall, findings from the two studies suggest that an eHealth tool, *HeadGear Apprentice*, was an acceptable and well-received intervention when adapted to young apprentices. However, questions remain regarding how to improve engagement and adherence to the program. These questions appear critical to effectiveness. The two studies also have implications for awareness raising in this population. Whilst preliminary results were encouraging, these improvements, along with a full-scale efficacy trial, are needed to better understand the utility of smartphone applications for mental health in this population.

**Trial registration:** ACTRN12618001475235 https://www.anzctr.org.au/Trial/Registration/TrialReview.aspx?id=375875&isReview=true.

## Introduction

Most mental health disorders emerge prior to the age of 25 years (1). The 2007 National Survey of Mental Health and Well-being found the prevalence of 12-months mental disorders was highest in young people aged 16–24 years, but service use was also the lowest (2). As such, there is a growing focus on prevention programs to reduce the incidence of new episodes of mental disorders by managing risk factors, enhancing resilience (3), and the relaying of personal risk information (4).

The transition from school to work is a unique developmental challenge that presents an opportunity for intervention. Some of the most well-described modifiable risk factors for common mental disorder are based in the workplace (5), yet relatively little attention has been devoted to young people as workers. Apprenticeships are a common pathway for young people making the transition from adolescence to adulthood and offer a prime opportunity for the delivery of mental health interventions. Furthermore, working conditions for some apprentices present significant challenges to mental wellness (6), and are implicated in heightened risk for anxiety, depression or stress disorder compared to older workers (7).

Mental health programs delivered online (eHealth) and via mobile technology (mHealth) can overcome barriers to young people receiving mental health information and support, as these modalities are provided in a practical, anonymous, and cost-effective manner (8). Our project team has developed a smartphone app (*HeadGear*) to help improve the mental health and well-being of workers in male-dominated industries (9). *HeadGear* involves a risk-profiling tool and a tailored 30-days mental health challenge to reduce risk, embodying evidence-based approaches, such as behavioral activation and mindfulness. In a large scale RCT, the app was found to reduce depression symptoms and prevent incident depression caseness (10). There was a specific dose-response effect present, with users, on average, completing one-third of the intervention content.

There is potential to adapt effective digital mental health interventions for specific populations to improve relevance and engagement (11). It is suggested that low engagement in mental health apps may be due to poor usability and lack of user-centric design (12). Meanwhile, there is support that tailored interventions, such as culturally adapted interventions, increase efficacy and reduce attrition (13). Yet there is little research to guide the adaptation of existing mental health interventions and subsequent evaluation for specific populations.

The aim of the current paper is to: (1) qualitatively evaluate the *HeadGear* app within an apprentice population; and (2) evaluate the usability, acceptability, feasibility, and preliminary efficacy of a modified version of the app (*HeadGear Apprentice*), designed to reduce depressive symptoms in an apprentice working population.

## Method

### Study 1: Focus Testing to Qualitatively Evaluate the *HeadGear* App

#### Participants and Recruitment

Registered group training organizations in Sydney and Newcastle, Australia, promoted the study to apprentices through their communication channels which included emails, flyers, and class announcement notices. The promotional material invited apprentices to take part in focus groups to explore how to support apprentice mental well-being. Interested participants registered with an onsite training group coordinator. To be included in the focus groups participants had to be enrolled in an apprenticeship program, fluent in English language, and a resident of Australia.

#### Procedure

This study formed part of a larger qualitative study of 54 apprentices (across eight activity-based focus groups), with a subset taking part in this component (*N* = 26) during September to November 2017. The overall sample for Study One was derived from a larger qualitative study (*N* = 54). These focus groups were randomly split (via block randomization) at each focus group occasion. This manuscript reports on those randomized to review the app, while the other half of the overall sample explored the concept of risk assessment and the reporting of risk. Participants gave written consent and completed demographic questionnaires at the beginning of all focus groups. In neither Study One nor Study Two were participants were not asked to disclose their employers. Additionally, it was made clear to participants that no findings would be directly shared with employers, and all published data would be at a deidentified (aggregated) level.

Each session was conducted by two researchers and lasted for ~90 min. A semi-structured discussion guide was used. The initial stage explored the challenges (6) and supports (14) used by apprentices; participants then spent the remainder of the group exploring the *HeadGear* app, discussing it, and completing questionnaires. All participants were reimbursed with a $40 gift card for their time.

This research was approved by the Human Research Ethics Committee at the University of Sydney (2017/648).

#### Intervention

*HeadGear* is a smartphone application-based intervention centered on behavioral activation and mindfulness therapy. The main therapeutic component of the *HeadGear* app takes the form of a 30-days challenge in which users complete one “challenge” daily. These include psychoeducational videos; mindfulness exercises; value-driven activity planning, goal setting, and review; and coping skill development (problem solving, sleep, grounding, alcohol use, assertiveness, and training in adaptive forms of coping). Incorporated into the app was a risk calculator, which assessed and provided participants with personalized feedback regarding their risk for future mental health issues. The risk calculator was developed from the validated HILDA risk algorithm for future distress in working Australian adults (15). The risk factor items are based on participant self-report. Other components of the app include a mood monitoring widget, a toolbox of skills (which is built from the challenge as it is completed), and support service helplines. The app was developed following a model of user engagement involving workshops, focus testing, and surveys with a range of relevant end users and stakeholders (9, 16, 17).

#### Measures

The self-report uMARS Scale (18) provides comprehensive ratings of user experience and impressions of the app by assessing app quality (objective and subjective) and perceived impact. Each item has customized wording appropriate to the aspect being assessed. Items employ a common 5-point rating scale from 1 (*Inadequate*) to 5 (*Excellent*), such that higher scores represent a stronger impact of the app on that aspect of user cognition and/or potential behavior. The subjective quality and perceived impact of particular app features were rated under each subscale of objective App Quality, assessed on an individual basis per item. Overall objective quality was measured using mean subscale scores.

#### Data Analysis

Study one formed part of a larger qualitative study. The prevailing theory is that sample size is based on the concept of “saturation” (i.e., sufficiently describe the phenomenon of interest, and address the research question at hand). Recently the idea of “information power” (the more information the sample holds, relevant for the actual study, the lower amount of participants is needed) has been proposed to estimate saturation (19). Although a number of elements must be considered in this definition, our focus was on the gleaning of new information from this group broadly, which we determined to be achieved as the later groups failed to present significantly new information.

No formal inferential analysis was undertaken on this data, descriptive statistics are reported pertaining to the uMARS. User feedback is also reported and informed Study Two.

### Study 2: Pilot Trial to Evaluate the Adapted *HeadGear* Apprentice App

#### Participants and Recruitment

Participants were recruited via three methods: (1) email circulation and snowball recruitment within industry partner organizations; (2) recruitment flyers, email, and site visits with education partner organizations; and (3) social media advertising. Eligible participants were required to be aged between 16 and 30 years, an Australian resident, fluent in English, enrolled in an apprenticeship program; and to have a valid email address and mobile number, and own an Apple- or Android-operating smartphone.

#### Procedure

Trial promotion materials directed interested participants to the trial website, upon which screening took place and consent was obtained electronically, between March and May 2019. After completing the online questionnaire battery, participants were directed to their respective app store to download the app.

**Figure 1:**
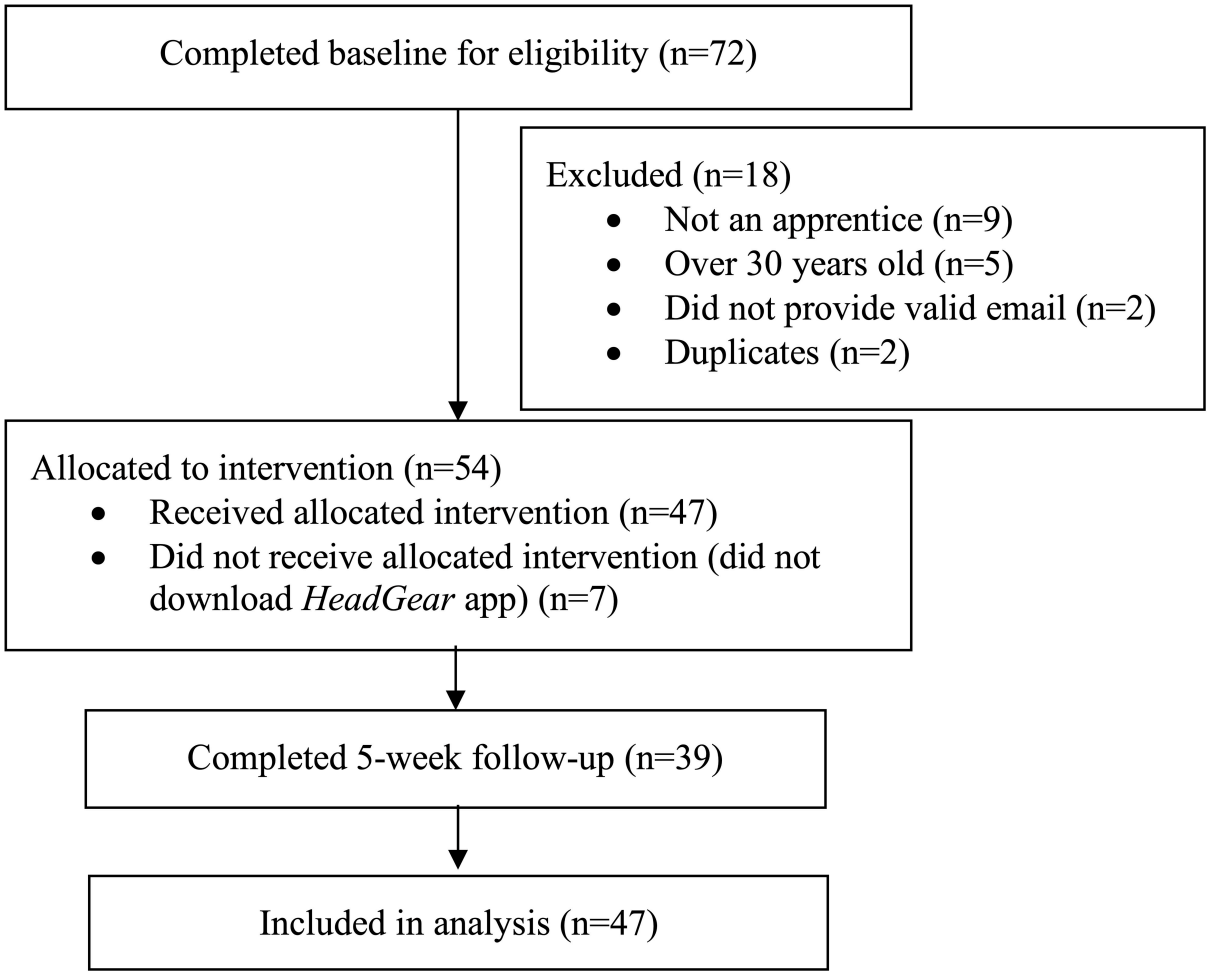
Flow of users through the trial.

Participants were encouraged to use the *HeadGear* apprentice app for 30 days. Objective app usage data was collected in-app. At 5-weeks post-baseline, participants were directed via email and SMS to complete the follow-up survey online. Participants were also invited to complete a telephone interview regarding their use of the app, and a 3-month online follow-up survey using the same measures completed at the 5-weeks assessment. The flow of users through the trial is presented in Figure 1.

This research was approved by the Human Research Ethics Committee at the University of Sydney (2018/788).

#### Intervention

Following on from the focus group testing (Study One), minor modifications were made to the *HeadGear* app including some changes to wording to increase accessibility, orientation video and improved navigation, specific apprentice support service guidance, the ability to skip through certain challenges and elements to enhance gamification (including badges for achievements). Personalization was also added to the risk assessment tool, directing users to the challenge days which were deemed to be most relevant based on their scores.

#### Outcome Measures

Participants completed self-administered questionnaires online. Demographic information provided included age, sex, education, area of study, year of apprenticeship, current medication, and help-seeking. They also completed the questionnaires outlined below.

Patient Health Questionnaire (PHQ-9) (20): The PHQ-9 is a reliable and valid nine-item measure of depression severity over the past 2 weeks and is sensitive to change (21, 22). The PHQ-9 can be used either as a diagnostic algorithm to make a probable diagnosis of major depressive disorder (MDD) or as a continuous measure with scores ranging from 0 to 27 and cut-points of 5, 10, 15, and 20 representing mild, moderate, moderately severe and severe levels of depressive symptoms (Cronbach's α = 0.89).

General Anxiety Disorder-7 item (GAD-7) (23): The GAD-7 is a reliable and valid seven-item measure of generalized anxiety symptoms, and it has also proved to have good sensitivity and specificity as a screening measure for panic, social anxiety, and post-traumatic stress disorder (24). GAD-7 scores can range from 0 to 27, with 5, 10, and 15 representing mild, moderate and severe levels of anxiety symptoms [Cronbach's α = 0.89 (25)].

The 5-item World Health Organization Well-Being Index (WHO-5) (26): Scores on the WHO-5 range from 0 to 25 where 0 indicates the worst possible quality of life and a score of 25 represents the best possible quality of life. A score ≤13 or an answer of 0 or 1 on any of the five items shows poor well-being. The WHO-5 is a psychometrically sound measure of well-being with high internal consistency (Cronbach's α = 0.84) and convergent associations with other measures of well-being (27).

Work performance was measured using three items from the Health and Work Performance Questionnaire (HPQ) (28) and an additional item pertaining to days absent in the last month. For the purposes of analysis, a composite measure for effective work days was constructed, by multiplying days present at work (absenteeism) by absolute work productivity score (presenteeism) as calculated by the HPQ, replicating previous work in the area (29).

The follow-up survey comprised of the same measures as in the initial battery with the addition of an adapted version of the Usefulness, Satisfaction, and Ease questionnaire (30), which has been used successfully in previous research (9). Participants were asked to rate their agreement with a series of statements about the intervention. Usage data was automatically collected by the app including time spent in app, number of logins, number of challenges completed, and specific responses to exercises.

#### Data Analysis

In the previous study using the *HeadGear* app (9), a small to moderate within group effect size (28) was observed. Power calculations showed that 44 participants would be needed to achieve this effect size with 80% power at alpha = 0.05. To account for an expected 30% dropout rate, 63 participants were to be recruited.

All data was analyzed using SPSS version 23.0. Descriptive statistics regarding participant characteristics and smartphone use data were analyzed to characterize engagement and acceptability. Paired samples *t*-tests were used to test for differences between pre- and post-trial clinical outcomes (e.g., PHQ-9). No adjustments were made for missing data; however, a sensitivity analysis was carried out utilizing last observation carried forward, with no differences found. To explore impact of actual intervention component exposure on symptoms and the effect of engagement, usage was segmented in tertiles based on overall use. All *p*-values were two-sided (one-sided for the *t*-test), with significance set at 5%. Effect size (*Cohen's d*) was calculated using mean change/baseline SD (31).

A series of open-ended interviews were conducted, via telephone, to ascertain themes and/or patterns pertaining to participants' evaluation of the *HeadGear* app for apprentices. Consistent with methods for the analysis of generative participatory data (32), an inductive approach to thematic analysis was taken with the transcripts of audio interviews (33–35). Coding was conducted independently by a researcher not involved in initial interviews (IL). Independently, a second researcher reviewed the recordings. Researcher codes were compared, and consensus reached via comparison and discussion (where needed) (34).

## Results

### Study One

#### Sample Characteristics

Typical of this target group, apprentices were aged 18–30 years, male (100%), and all owned a smartphone with approximately half iPhone and half Android type. The majority (83%) were undertaking a full-time apprenticeship and were completing an electronics or construction-related trade (Table 1).

**Table 1:** Sample characteristics of focus group participants (*N* = 26).

	***n* (%)**
Mean age (SD)	20.77 (3.0)
**GENDER**	
Male	26 (100%)
**SMARTPHONE OWNED**	
Android	10 (38.5)
iPhone	15 (57.7)
Other (Google, Windows)	1 (3.8)
**LENGTH OF TIME IN APPRENTICESHIP**	
<1 year	8 (30.8)
1–2 years	13 (50.0)
3–4 years	4 (15.4)
**TYPE OF APPRENTICESHIP**	
Full-time	24 (92.3)
Part-time	1 (3.8)
School-based	1 (3.8)
**AREA OF STUDY**	
Commercial cookery/hospitality	2 (15.4)
Electronics	9 (34.6)
Construction trade (plumbing, bricklaying, carpentry, electrician)	11 (42.3)
Other	1 (3.8)
**LOCATION OF APPRENTICESHIP**	
Metropolitan	18 (69.2)
Regional	8 (30.8)

#### App Quality

Overall, apprentices rated the *HeadGear* app positively, with an average of 4/5 stars (Table 2). Ratings for objective quality altogether indicated good objective quality (3.8) and were similar across all aspects indicating consistent degree of quality in terms of all features. Specifically, “customization” under Engagement was poorest (2.8) whilst “layout” in “Aesthetics” was rated highest, closely followed by credibility and quality of information. While most apprentices would widely recommend the app (all endorsing on average a “likely” recommendation to at least several individuals), on average users predicted their use would be infrequent (3–10 times) over the next 12 months, unlikely to allow the app to have sufficient therapeutic impact. Encouragingly though, around one-third expressed interest in more frequent use 10–50 times in the next year. Participants generally had no or neutral willingness to pay for the app, with 65.4% not at all. On average, apprentices reported that the *HeadGear* app had a consistent moderate degree of impact upon their awareness, knowledge, attitudes, intention to change, help-seeking, and behavior change around mental health and well-being.

**Table 2:** uMARS subscale ratings.

**Subscale**	**Score**
App objective quality	*M* (*SD*); min–max
A. Engagement	3.6 (0.47); 2.8–4.6
B. Functionality	3.9 (0.49); 3–4.8
C. Aesthetics	4.1 (0.66); 3–5
D. Information	4.1 (0.66); 3–5
Overall objective quality	3.8 Good (0.46); 3.1–4.8
App Subjective Quality	Rating (score)
Recommend the app to others	Probably (4)
Predicted frequency of use of app in next year	3–10 (3)
Willing to pay for the app	Probably not (2)
Overall star rating	• • • • (4/5 stars)
Perceived impact of app (*N* = 26)	*M* (*SD*); min–max
Awareness	3.9 (0.72); 2–5
Knowledge	3.8 (0.90); 2–5
Attitudes	3.9 (0.93); 2–5
Intention to change	3.6 (0.90); 1–5
Help-seeking	3.9 (0.74); 2–5
Behavior change	4.0 (0.96); 1–5
Mean perceived impact for all factors	Moderate impact (4)

#### Feedback

The inability to skip challenges was raised as a negative point in terms of app engagement, highlighted by the low scores for customization. Participants sought the ability to pick and choose specific activities rather than progress through the challenge in linear succession. They also emphasized the importance of gamification and greater personalization within the app, for example through the inclusion of music. Participants also suggested minor changes to language used in the app.

### Study Two

Overall, 54 eligible participants consented to the study, of which 47 completed baseline assessment and downloaded the app. The characteristics of this sample are presented in Table 3. The sample was predominately male (96%), with a mean age of 21.7 years. Participants' apprenticeship experience was relatively evenly spread, with the majority working in the areas of building and construction, electronics, and engineering. On average participants scored in the mild range for depression and anxiety at baseline; however, 15% were currently seeking mental health support.

**Table 3:** Sample characteristics of pilot app evaluation participants (*N* = 47).

	***n* (%)**
Mean age (SD)	21.68 (3.62)
**BASELINE MEAN SCORES**	
PHQ-9 (SD)	7.06 (5.54)
GAD7 (SD)	5.94 (5.03)
WHO5 (SD)	13.55 (5.20)
**GENDER**	
Male	45 (96.7)
**APPRENTICESHIP YEAR**	
1st	14 (29.8)
2nd	16 (34.0)
3rd	11 (23.4)
4th	6 (12.8)
**EMPLOYMENT**	
Full-time	47 (100.0)
**AREA OF STUDY**	
Electrical and electronics	16 (34.0)
Carpentry/joinery/cabinet making	14 (29.8)
Engineering and machinery	7 (14.9)
Hospitality and Cookery	4 (8.5)
Plumbing	3 (6.4)
Automotive trades and services	2 (4.3)
**EDUCATION**	
Year 10 certificate	9 (19.1)
Year 12 certificate	29 (61.7)
Trade or other certificate	5 (10.6)
University degree	4 (8.5)
**GROUP TRAINING ORGANIZATION**	
TAFE	32 (68.1)
Other provider	15 (31.9)
Current mental health help	7 (14.9)
Current medication	2 (4.3)

#### App Usage and Feedback

On average users spent 77.4 (*SD* = 59.30) min in the app, over 21 (median) sessions. Users completed approximately a third of the app challenges (*M* = 11.91; *SD* = 11.25). The app was well-received by the participants, with 87.2% claiming it had at least moderately improved their mental fitness. The majority understood the app content (87.2% very/completely), while three-quarters (74.4%) claimed they would probably/definitely recommend the app to others. The appropriateness of app content had slightly lower appeal (66% very/completely), while app engagement was slightly lower again (59% very/completely). Overall, participants rated the app highly or very highly (74.4%).

#### Interviews

While eleven participants agreed to be interviewed, only four could be reached (8.5% of participants overall). Of the emergent themes, there was a consensus as to the positive overall impact of the app on mental health, significance of tailoring the app to apprentices specifically, importance of reminders, and a failure to make use of the toolbox function. Nonetheless, the participants shared different viewpoints in relation to technical difficulties, general usage of the app, and value of daily challenges; specifically, the mindfulness, mood monitoring, and action-planning/goal setting activities. Two of these users completed the entire challenge, with the remaining interviewees completing 7 and 20 challenges, respectively.

Firstly, it was unanimously indicated that the *HeadGear* for apprentices app elicited positive effects on their mental health (e.g., “*the app in general is really good”* and helped the user “*to think about a lot of things [he] doesn't end up thinking about during the day”*). Consistently, reasons for participation were driven by the tailoring of the app to apprentices specifically (e.g., “*I think that it's good it's targeted to apprentices… they might not always seek out other mental health apps”*).

With respect to app usage, non-completion of challenges was mainly attributed to “*forgetting”* to do so and then choosing not to “*catch up”*. Participants consistently reported that the reminders were “*helpful”*; however, they also reported that these could be improved on by allowing the user to select the time of the reminder notification (this was possible within the app although participants were not aware of this function). Specifically, presenting reminders were “at lunch time or 4 p.m. when he finishes work” was suggested.

Several interviewees stated that they did not (or rarely) utilize the toolbox function, citing unawareness, forgetfulness, and laziness as reasons.

Although one respondent found the goal setting exercises to be the most helpful of the challenges (“the goal setting and tracking was very helpful… it helped me to stay on top of it and things I needed to do.”), in general there was a reluctance to engage in action planning (e.g., “*mental exercises were better than the active exercises,” [I disliked] activities associated with “list[s] and planning”*).

The mindfulness challenges were well-received (“*very helpful technique I learnt”*), as were the psychoeducational videos which were described as being “*helpful and fun to watch.”* In both cases the inclusion of a transcript was considered important.

The interviewees expressed divergent opinions when discussing the efficacy of the mood monitoring function. Where it was not used, interviewees asserted that they felt as though their “*moods [don't] really change from being genuinely happy so [I] didn't think I needed to track it”*. Conversely, others found it incredibly useful, and continued to use it “*most days”, “at the end of the day to see how I feel [about] the day as a whole”* claiming that it enabled them to “look at it over a month and go… was it just a sh^*^tty month? Was it a sh^*^tty stuff up thing or has my outlook perhaps changed?”

#### Symptom Levels and Productivity

Overall, 39 participants (82.9%) completed follow-up questionnaires. Symptom levels were generally in the mild range at follow-up. There was a positive trend across all the outcomes of interest over the 5-weeks follow-up; however, only well-being (WHO-5) reached statistical significance (Table 4).

**Table 4:** Change in outcome scores over time.

	**Pretrial mean (SD)**	**Post-trial mean (SD)**	***F* (df)**	**Significance**	**Effect size (*Cohen's d*)**
PHQ-9	6.74 (5.47)	5.26 (4.35)	3.777 (1.38)	0.059	0.27 (−0.16–0.69)
WHO-5	13.36 (5.09)	14.85 (5.70)	4.204 (1.38)	0.047	−0.29 (−0.72–0.14)
GAD-7	6.08 (5.41)	4.95 (3.69)	2.633 (1.38)	0.113	0.21 (−0.24–0.65)
Effective work days	18.98 (3.99)	19.55 (5.14)	0.772 (1.38)	0.385	−0.14 (−0.59–0.30)

**Figure 2:**
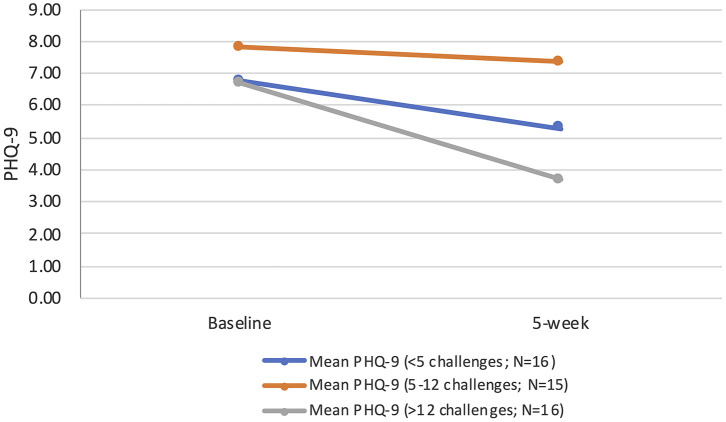
Dose-response by tertiles.

When exploring the impact of actual intervention component exposure on the main outcome of interest (depression symptoms), one third completed fewer than five challenges, one-third completed 5–12 and the remaining third completed >12. Those completing more than 12 sessions had significantly reduced depressive symptoms at 5-weeks follow-up [*F*_(1,15)_ = 11.25; *p* = 0.004; mean difference: 3.00], whereas those who completed fewer than 5, or 5–12 sessions, showed no significant difference. Dose-response of the intervention by tertiles are presented in Figure 2.

#### 3-Month Follow-Up

Of the 47 eligible participants, 19 (40%) completed 3-month follow-up. There were no significant differences between baseline and 3-month follow-up across outcomes of interest. Similarly, there were no significant differences between scores at the two follow-up time points. Low questionnaire completion precluded the exploration of app usage at this timepoint.

## Discussion

This paper aimed to explore the utility of a mental health app, *HeadGear*, within an apprentice population. To the authors' knowledge, this is the first time a tailored eHealth program has been developed for this population. Overall, the app was well-received in both studies; however, engagement (measured through both self-report and intervention adherence) was an issue. This is unsurprising due to the difficulties in engaging this group in mental health interventions generally (36). Nevertheless, this had implications for the preliminary effectiveness of the app.

Previous trials of mental health apps with young people highlight the difficulties in achieving program adherence within this population (37). Despite minimal feedback on ways to improve the app at the conclusion of Study One, the incorporation of elements to improve engagement and completion rates are required, highlighted by the links between completion and symptom change highlighted in Study Two. The results of this paper highlight the importance of engagement, and that simply relying on positive subjective reports of usability, satisfaction, acceptability, or feasibility is insufficient when determining actual engagement (38). Peter's et al. (39) suggest that the psychological needs of autonomy, competence and relatedness mediate positive user experience outcomes including engagement and may be critical factors in designing interventions. This is in-line with some of the qualitative feedback including seeking customization (autonomy), gamification (competence satisfaction), and language (relatedness).

Furthermore, the results suggest that engagement (in the form of intervention completion) is directly related to effect of an app-based intervention of this kind. Although it has been shown that completion of digital mental health interventions is not always directly correlated with outcomes (38), and the minimum level of engagement required for such interventions to achieve beneficial effects remains an open question (40, 41). Involving end-users in the conception, design, and implementation of any app is a critical component to successful design (18)—in the case of *HeadGear*, this was a core component of development (6, 14). Indeed many recommendations to enhance engagement in this young, predominately male population (36) were incorporated in the preliminary work completed as part of the app modifications. Other useful techniques to enhance engagement may include a means to better embed the technology within the systems and structures which users already operate within (e.g., clinical services) (18); in this case, given the low levels of traditional service use within the apprentice population, workplaces or training organizations may place a vital role. This may also be a means to provide supportive accountability, which is linked to enhanced engagement (42). However, there remains the question of how to create habit-forming technologies within this space by improving the intrinsic motivation to complete such programs, which requires intensive multidisciplinary development.

Symptom change is, indeed, only one element of importance in developing programs within this population. The apprenticeship experience and support given to mental health issues can vary greatly, especially in small employers (43). Importantly, the response to the app was positive in terms of perceived impact on awareness, help-seeking, and behavior, along with subjective mental fitness. Such elements are critical to adequately serving a population that has low levels of mental health literacy (44) and high rates of training incompletion, with factors related to poor mental health reported as the primary reasons for incompletion (45).

Although only well-being scores improved to a statistically significant degree, there was a consistent trend toward improvements across all health and behavioral outcomes. The true potentially beneficial impact of the app may be obscured in this study due to lack of power and low baseline symptom rates (10). Furthermore, the app was designed to prevent rather than treat depression and in a pilot trial of this kind exploring such an outcome was not feasible. This is a limitation of the current study, and requires much greater numbers to determine effectiveness (46). Nevertheless, baseline depression scores and app usage were comparable within this sample and the original *HeadGear* prevention trial (10). In the original trial users completed on average 9 challenges, using the app for 52 min. The within-group effect size in the current study was slightly smaller at post-intervention (0.8 vs. 1.2), but the mean difference was similar (1.80 vs. 1.94). Comparatively, well-being scores improved to a greater extent within the current trial (−0.41 vs. −0.62).

Other limitations of the study include the low rates of female participation in both studies; while this is reflective of the makeup of industries sampled, there remains a question around whether female apprentices would respond differently to this app than males. Similarly, the range of industries represented was limited, and findings may not be generalizable to all apprentices. Finally, as with any study of this kind there is the potential for self-report bias, nevertheless, evidence suggests self-report provides useful and accurate estimates when conditions are designed to maximize response accuracy (47, 48). To minimize bias in responding we intentionally recruited via non-workplace channels (e.g., training organizations and online). Overall, the findings from the two studies reported here suggest that an eHealth tool, the *HeadGear* application, was generally considered acceptable and well-received when adapted to young apprentices. However, questions remain regarding how to improve engagement and adherence to the program. These questions appear critical to effectiveness. Whilst preliminary results were encouraging, these improvements, along with a full-scale efficacy trial are needed to better understand the utility of smartphone applications for mental health in this population.

## Data Availability Statement

The datasets presented in this article are not readily available because ethics approval states no individual participant results or identification will be published or accessed by anyone other than the research team. Requests to access the datasets should be directed to Mark Deady, m.deady@unsw.edu.au.

## Ethics Statement

The University of Sydney Human Research Ethics Committee approved these studies (2018/788, 2017/648). All participants gave written informed consent before data collection began.

## Author Contributions

MD, DC, IL, IC, and SH had full access to all the data in the study and take responsibility for the integrity of the data. MD had a primary role in conceptualization and write up and editing of this manuscript. IC had a secondary role in conceptualization and write up and editing of this manuscript. NG and SH had a role in conceptualization and editing of this manuscript. DC, IL, AG, HC, and IC had a role in write up and editing of this manuscript. AW and RE had a role data collection and editing. RC had a role in technical development of the intervention and editing of this manuscript. HC had a role in write up and editing of this manuscript. All authors have read and approved of the final manuscript.

## Conflict of Interest

MD, IC, NG, RC, and SH were involved in the development of the HeadGear application. The IP was jointly owned by MD, IC, NG, RC, and SH, however, the authors do not currently receive any financial gain from this IP. The remaining authors declare that the research was conducted in the absence of any commercial or financial relationships that could be construed as a potential conflict of interest.

## References

[1] McGorry PD Purcell R Goldstone S Amminger GP. Age of onset and timing of treatment for mental and substance use disorders: implications for preventive intervention strategies and models of care. Curr Opin Psychiatry. (2011) 24:301–6. 10.1097/YCO.0b013e3283477a09 21532481

[2] Slade T Johnston A Oakley Browne MA Andrews G Whiteford H. 2007 National survey of mental health and wellbeing: methods and key findings. Aust Nz J Psychiat. (2009) 43:594–605. 10.1080/00048670902970882 19530016

[3] Cuijpers P Van Straten A Smit F. Preventing the incidence of new cases of mental disorders: a meta-analytic review. J Nerv Ment Dis. (2005) 193:119–25. 10.1097/01.nmd.0000152810.76190.a6 15684914

[4] Bellón JÁ Conejo-Cerón S Moreno-Peral P King M Nazareth I Martín-Pérez C. Intervention to prevent major depression in primary care: a cluster randomized trial preventing major depression in primary care. Ann Intern Med. (2016) 164:656–65. 10.7326/M14-2653 27019334

[5] Henderson M Harvey SB Øverland S Mykletun A Hotopf M. Work and common psychiatric disorders. J R Soc Med. (2011) 104:198–207. 10.1258/jrsm.2011.100231 21558098 PMC3089873

[6] Einboden R Choi I Ryan R Petrie K Johnston DA Wray A ‘Having a thick skin is essential’: mental health challenges for young apprentices in Australia. J Youth Stud. 10.1080/13676261.2020.1728240. [Epub ahead of print].

[7] Safe Work Australia Work-related mental disorders profile. Canberra, ACT: Safe Work Australia (2015).

[8] Ellis LA McCabe KL Rahilly KA Nicholas MA Davenport TA Burns JM Encouraging young men's participation in mental health research and treatment: perspectives in our technological age. Clin Invest. (2014) 4:881–8. 10.4155/cli.14.61

[9] Deady M Johnston D Milne D Glozier N Peters D Calvo R Feasibility, acceptability and preliminary effectiveness of a smartphone-app to reduce depressive symptoms in the workplace. JMIR mHealth. (2018) 3:e11661 10.2196/11661 PMC6299234 30514694

[10] Deady M Glozier N Calvo RA Johnston DA Mackinnon A Milne D. Preventing depression using a smartphone app: a randomised clinical trial. Psychol Med. 10.1017/S0033291720002081. [Epub ahead of print]. 32624013

[11] Schueller SM Hunter JF Figueroa C Aguilera A Use of digital mental health for marginalized and underserved populations. Curr Treat Opt Psychiatry. (2019) 6:243–55. 10.1007/s40501-019-00181-z

[12] Torous J Nicholas J Larsen ME Firth J Christensen H. Clinical review of user engagement with mental health smartphone apps: evidence, theory and improvements. Evid Based Mental Health. (2018) 21:116–9. 10.1136/eb-2018-102891 29871870 PMC10270395

[13] Harper Shehadeh M Heim E Chowdhary N Maercker A Albanese E. Cultural adaptation of minimally guided interventions for common mental disorders: a systematic review and meta-analysis. JMIR Mental Health. (2016) 3:e44. 10.2196/mental.5776 27670598 PMC5057065

[14] Choi I Petrie K Einboden R Collins D Ryan R Johnston D Exploring young male workers' healthy coping strategies and attitudes to using a smartphone app to support their mental health (in press).

[15] Fernandez A Salvador-Carulla L Choi I Calvo R Harvey SB Glozier N Development and validation of a prediction algorithm for the onset of common mental disorders in a working population. Aust N Z J Psychiatry. 2017:0004867417704506 10.1177/0004867417704506 28403625

[16] Deady M Peters D Lang H Calvo R Glozier N Christensen H. Designing smartphone mental health applications for emergency service workers. Occup Med. (2017) 67:425–8. 10.1093/occmed/kqx056 28535246

[17] Peters D Deady M Glozier N Harvey S Calvo RA. Worker preferences for a mental health app within male-dominated industries: participatory study. JMIR Ment Health. (2018) 5:e30. 10.2196/mental.8999 29695371 PMC5943624

[18] Stoyanov SR Hides L Kavanagh DJ Wilson H. Development and validation of the user version of the mobile application rating scale (uMARS). JMIR Mhealth Uhealth. (2016) 4:e72. 10.2196/mhealth.5849 27287964 PMC4920963

[19] Malterud K Siersma VD Guassora AD. Sample size in qualitative interview studies: guided by information power. Qual Health Res. (2015) 26:1753–60. 10.1177/1049732315617444 26613970

[20] Kroenke KMD Spitzer RLMD The PHQ-9: a new depression diagnostic and severity measure. Psychiatric Ann. (2002) 32:509–15. 10.3928/0048-5713-20020901-06

[21] Löwe B Kroenke K Herzog W Gräfe K. Measuring depression outcome with a brief self-report instrument: sensitivity to change of the patient health questionnaire (PHQ-9). J Aff Disord. (2004) 81:61–6. 10.1016/S0165-0327(03)00198-8 15183601

[22] Martin A Rief W Klaiberg A Braehler E. Validity of the brief patient health questionnaire mood scale (PHQ-9) in the general population. Gen Hosp Psychiatry. (2006) 28:71–7. 10.1016/j.genhosppsych.2005.07.003 16377369

[23] Spitzer RL Kroenke K Williams JBW Löwe B. A brief measure for assessing generalized anxiety disorder: the GAD-7. Arch Intern Med. (2006) 166:1092–7. 10.1001/archinte.166.10.1092 16717171

[24] Kroenke K Spitzer RL Williams JBW Löwe B The patient health questionnaire somatic, anxiety, and depressive symptom scales: a systematic review. Gen Hosp Psychiatry. (2010) 32:345–59. 10.1016/j.genhosppsych.2010.03.006 20633738

[25] Löwe B Decker O Müller S Brähler E Schellberg D Herzog W. Validation and standardization of the generalized anxiety disorder screener (GAD-7) in the general population. Med Care. (2008) 46:266–74. 10.1097/MLR.0b013e318160d093 18388841

[26] Topp CW Østergaard SD Søndergaard S Bech P. The WHO-5 well-being index: a systematic review of the literature. Psychother Psychosom. (2015) 84:167–76. 10.1159/000376585 25831962

[27] Bech P Measuring the dimensions of psychological general well-being by the WHO-5. QoL Newsletter. (2004) 32:15–6.

[28] Kessler RC Barber C Beck A Berglund P Cleary PD McKenas D. The world health organization health and work performance questionnaire (HPQ). J Occup Environ Med. (2003) 45:156–74. 10.1097/01.jom.0000052967.43131.51 12625231

[29] Wang PS Simon GE Avorn J. Telephone screening, outreach, and care management for depressed workers and impact on clinical and work productivity outcomes: a randomized controlled trial. JAMA. (2007) 298:1401–11. 10.1001/jama.298.12.1401 17895456 PMC2859667

[30] Lund A Measuring usability with the USE questionnaire. Usabil User Exp. (2001) 8:8.

[31] Morris SB DeShon RP. Combining effect size estimates in meta-analysis with repeated measures and independent-groups designs. Psychol Methods. (2002) 7:105–25. 10.1037/1082-989X.7.1.105 11928886

[32] Sanders L Stappers PJ Convivial Toolbox: Generative Research for the Front End of Design. Amsterdam: BIS (2013).

[33] Braun V Clarke V Using thematic analysis in psychology. Qual Res Psychol. (2006) 3:77–101. 10.1191/1478088706qp063oa

[34] Coffey A Atkinson P Making Sense of Qualitative Data: Complementary Research Strategies. Thousand Oaks, CA: Sage Publications (1996).

[35] Saldaña J The Coding Manual for Qualitative Researchers. London: Sage Publications Ltd. (2015).

[36] Ellis LA Collin P Hurley PJ Davenport TA Burns JM Hickie IB. Young men's attitudes and behaviour in relation to mental health and technology: implications for the development of online mental health services. BMC Psychiatry. (2013) 13:119. 10.1186/1471-244X-13-119 23601273 PMC3651363

[37] Bohleber L Crameri A Eich-Stierli B Telesko R von Wyl A Can we foster a culture of peer support and promote mental health in adolescence using a web-based app? A control group study. JMIR Ment Health. (2016) 3:e45 10.2196/mental.5597 27663691 PMC5074648

[38] Donkin L Hickie IB Christensen H Naismith SL Neal B Cockayne NL. Rethinking the dose-response relationship between usage and outcome in an online intervention for depression: randomized controlled trial. JMIR. (2013) 15:e231. 10.2196/jmir.2771 24135213 PMC3806549

[39] Peters D Calvo RA Ryan RM. Designing for motivation, engagement and wellbeing in digital experience. Front Psychol. (2018) 9:797. 10.3389/fpsyg.2018.00797 29892246 PMC5985470

[40] Donkin L Christensen H Naismith SL Neal B Hickie IB Glozier N. A systematic review of the impact of adherence on the effectiveness of e-therapies. J Med Internet Res. (2011) 13:e52. 10.2196/jmir.1772 21821503 PMC3222162

[41] Bakker D Rickard N. Engagement in mobile phone app for self-monitoring of emotional wellbeing predicts changes in mental health: MoodPrism. J Affect Disord. (2018) 227:432–42. 10.1016/j.jad.2017.11.016 29154165

[42] Mohr DC Cuijpers P Lehman K. Supportive accountability: a model for providing human support to enhance adherence to ehealth interventions. J Med Internet Res. (2011) 13:e30. 10.2196/jmir.1602 21393123 PMC3221353

[43] Buchanan J Raffaele C Glozier N Kanagaratnam A Beyond Mentoring: Social Support Structures for Young Australian Carpentry Apprentices. Adelaide, SA: NCVER (2016).

[44] Jorm A. Mental health literacy. Empowering the community to take action for better mental health. Am Psychol. (2012) 67:231–43. 10.1037/a0025957 22040221

[45] Bednarz A Understanding the Non-Completion of Apprentices. Adelaide, SA: National Centre for Vocational Education Research (NCVER) (2014).

[46] Cuijpers P. Examining the effects of prevention programs on the incidence of new cases of mental disorders: the lack of statistical power. Am J Psychiatry. (2003) 160:1385–91. 10.1176/appi.ajp.160.8.1385 12900296

[47] Del Boca FK Noll JA. Truth or consequences: the validity of self-report data in health services research on addictions. Addiction. (2000) 95:347–60. 10.1046/j.1360-0443.95.11s3.5.x 11132362

[48] Del Boca FK Darkes J. The validity of self-reports of alcohol consumption: state of the science and challenges for research. Addiction. (2003) 98:1–12. 10.1046/j.1359-6357.2003.00586.x 14984237

